# Isolation, characterization, and genomic analysis of a lytic bacteriophage, PQ43W, with the potential of controlling bacterial wilt

**DOI:** 10.3389/fmicb.2024.1396213

**Published:** 2024-08-01

**Authors:** Binbin Huang, Long Ge, Dong Xiang, Ge Tan, Lijia Liu, Lei Yang, Yongfeng Jing, Qingshu Liu, Wu Chen, Ye Li, Haoxin He, Huzhi Sun, Qiang Pan, Ke Yi

**Affiliations:** ^1^China Tobacco Hunan Industrial Co., Ltd., Changsha, China; ^2^Hunan Provincial Engineering and Technology Research Center for Agricultural Microbiology Application, Hunan Institute of Microbiology, Changsha, China; ^3^Qingdao NABT PhagePharm Co., Ltd., Qingdao, China; ^4^College of Plant Protection, Hunan Agricultural University, Changsha, China; ^5^Institute of Special Food, Qingdao Agricultural University, Qingdao, China

**Keywords:** bacterial wilt (BW), *Ralstonia solanacearum*, *Ralstonia solanacearum* species complex (Rssc), bacteriophage, phage therapy

## Abstract

Bacterial wilt (BW) is a devastating plant disease caused by the soil-borne bacterium *Ralstonia solanacearum* species complex (Rssc). Numerous efforts have been exerted to control BW, but effective, economical, and environmentally friendly approaches are still not available. Bacteriophages are a promising resource for the control of bacterial diseases, including BW. So, in this study, a crop BW pathogen of lytic bacteriophage was isolated and named PQ43W. Biological characterization revealed PQ43W had a short latent period of 15 min, 74 PFU/cell of brust sizes, and good stability at a wide range temperatures and pH but a weak resistance against UV radiation. Sequencing revealed phage PQ43W contained a circular double-stranded DNA genome of 47,156 bp with 65 predicted open reading frames (ORFs) and genome annotation showed good environmental security for the PQ43W that no tRNA, antibiotic resistance, or virulence genes contained. Taxonomic classification showed PQ43W belongs to a novel genus of subfamily *Kantovirinae* under *Caudoviricetes.* Subsequently, a dose of PQ43W for phage therapy in controlling crop BW was determined: 10^8^ PFU*20 mL per plant with non-invasive irrigation root application twice by pot experiment. Finally, a field experiment of PQ43W showed a significantly better control effect in crop BW than the conventional bactericide Zhongshengmycin. Therefore, bacteriophage PQ43W is an effective bio-control resource for controlling BW diseases, especially for crop cultivation.

## 1 Introduction

Bacterial wilt (BW) is one of the most destructive diseases in many s*olanaceous* crops, such as potatoes, tobacco, tomatoes, pepper, and eggplant, and is a significant source of crop production loss worldwide (Mansfield et al., 2012; Jiang et al., 2017; Wang et al., 2023). The pathogen of BW, *Ralstonia solanacearum*, is a soil-borne bacterial pathogen and has long been part of the “*R. solanacearum* species complex” (Rssc) composed of three different *Ralstonia* species: *R. solanacearum*, *R. pseudosolanacearum*, and *R. syzygii* (Safni et al., 2014). The pathogen Rssc typically colonizes the root xylem tissues and infects the roots through small wounds, it then quickly moves to stem tissues and secretes a large amount of EPS, which causes xylem obstruction and leads to plant wilting (Jiang et al., 2017; Ingel et al., 2022). Many efforts have been engaged to control this disease (Yuliar et al., 2015; Mamphogoro et al., 2020; Wang et al., 2023), however, none of these strategies has been able to control the BW disease efficiently, due to the broad host range, lack of plant resistance cultivars, environmental pollution and genetic diversity of the pathogen, and so on (Mansfield et al., 2012; Safni et al., 2014). Therefore, how to effectively control BW remains an urgent challenge.

Bacteriophages (phages) are viruses that exclusively infect a certain bacterium and are not toxic to animals, plants, humans, or non-target microorganisms (Yuliar et al., 2015). They infect their host and exploit the metabolic processes of the host to replicate their own genome, thereby releasing more virions (Gordillo and Barr, 2019; Mamphogoro et al., 2020). So, phage therapy has attracted great attention and obtained good results, showing some advantages in the treatment of specific human or animal diseases, e.g., high lytic activity, specific targeting capabilities, cost-effectiveness, and no harm to the environment (Gordillo and Barr, 2019; Grabowski et al., 2022; Hatfull et al., 2022; Strathdee et al., 2023). Some research based on bacteriophages (phages) has shown the therapy’s great potential to control plant bacterium disease in crop cultivation (Balogh et al., 2018; Muturi et al., 2019; Marquioni et al., 2022; Villalpando-Aguilar et al., 2022). For example, phages research based on bacteriophages (phages) has shown great potential to control *Xanthomonas perforans* (Balogh et al., 2018). Phage vB_XciM_LucasX has been used to control citrus canker disease caused by *X. citri* and *X. fuscans* (Marquioni et al., 2022). Phage Wc5r has shown substantial results in preventing and controlling potato soft rot caused by *Pectobacterium carotovorum* (Muturi et al., 2019). Phages therapy to control crop bacterial diseases is also considered to be a promising measure (Álvarez and Biosca, 2017).

Interestingly, some *Ralstonia*-infecting bacteriophages have been isolated and achieved good results in corps cultivation against BW (Addy et al., 2018, 2019; Wang X. et al., 2019; Wang R. et al., 2019; Chen et al., 2023), such as *Aresaunavirus* of *Ralstonia* phage RsoM1USA, *Higashivirus* of *Ralstonia* phage RsoP1IDN, and *Gervaisevirus* of *Ralstonia* phage GP4. Due to the diversity of the geographic distribution of Rssc and the specificity of phage on host strains, it is very important to isolate more BW-pathogen infection phages to control the BW in crops cultivation, especially in crops with a high BW incidence rate (Wang X. et al., 2019; Liu et al., 2020; Ahmad et al., 2021; Kaur et al., 2021).

Due to the wide host nature of BW pathogen Rssc, we selected tobacco as our crop subject. Not only is tobacco is a typical Solanaceae crop and widely cultivated in China (Jiang et al., 2017), but there would also be great significance in controlling BW in tobacco cultivation as it would reduce the loss caused by BW in tobacco cultivation, reduce the BW-pathogen abundance in local agro-ecosystems, and act as a reference or guide to control BW in other crops cultivation. So, in this study, we report a lytic phage isolated from the rhizosphere soil of a tobacco BW disease plant which was collected in Hunan province of China. We characterized the morphology and biological characteristics and then sequenced and annotated the genome. We also explored the BW control efficacy of phages application in pot and field experiments. These studies are important for a better understanding of phages as a biocontrol resource against BW in crop cultivation.

## 2 Materials and methods

### 2.1 Isolation and cultivation of bacterial wilt-inducing pathogenic strains

Tobacco BW disease stem tissues were collected from a field with a high incidence rate for BW in Changsha (Hunan, China). Single colony isolation was performed according to the method employed by Okiro et al. (2022) with minor modifications. Stems with visible symptoms of bacterial wilt were collected, then, thoroughly cleaned with tap water, dried using paper towels, and sterilized with 70% ethanol. The stems were then crushed in sterile water for the bacteria to ooze from the stem tissues, and streaked in TTC media, followed by purification and identification of the bacteria. The strain was confirmed as the pathogen of tobacco BW disease by completing Koch’s rule according to the methods described by Huang et al. (2023). The isolated strain, named NdE, was stored in 25% (vol/vol) glycerol at −80°C and cultured for 16 h to 48 h at 30°C with 180 rpm shaking in nutrient broth medium (NB; Shanghai Sangon Biotech Co., Ltd., China).

The isolated strain was verified by 16S rRNA sequencing and blastn in NCBI Genbank. The phylogenetic tree based on the 16S rRNA sequence was produced by the neighbor-joining method in MEGA X, with 1,000 bootstrap replicates.

### 2.2 Bacteriophage isolation and purification

Bacteriophage (Phage) isolation was conducted according to the method described by Xu et al. (2021) with slight modifications. First, 1 g of fresh soil and 2 mL of host bacterial strains (OD_600_ = 0.5 to 1.0) were added into 100 mL sterile NB broth medium and co-cultured overnight at 28°C; the mixed culture was then centrifuged and filtered using 0.22 μm membranes to remove bacteria and to obtain phage stock solution. Secondly, the phage stock solution was used for double-layer plate assay at 100^-10^ dilution. Then, a single plaque was picked on a double plate and added to a new fresh host co-culture mixture for overnight culture, followed by 10,000 g, 5 min centrifugal and 0.22 μm filtration to purify the phage, and repeated three to five times to obtained the purified phages.

### 2.3 Morphology of the bacteriophage

Characterization of the phage morphology was conducted as described by Sasaki et al. (2021), with minor modifications. A fresh phage sample (10^9^ PFU/ml and 0.22 μm filtered) was visualized by transmission electron microscopy (TEM) using phosphotungstic acid (PTA) with negative staining. 20 μL of the liquid sample was dropped onto a copper mesh with a carbon-coated membrane, allowed to settle naturally for 15 min, dried on filter paper, and then stained with 2% (W/V) phosphotungstic acid (PTA) for 2 min. Excess liquid was absorbed by filter paper amd dried before being examined by transmission electron microscope (TEM) observation. More than 10 phage images were estimated and calculated with the morphometrics.

### 2.4 Biological characteristics

The biological characteristics of phage was conducted according to the methods of Tian et al. (2022) and Huang et al. (2022), described with minor modifications.

For temperature stability, the phage stock solution with a titer of 10^10^ PFU/ml was incubated at 30°C, 40°C, 50°C, 60°C, 70°C, and 80°C for 1 h, 2 h, and 3 h to determine the titers by the double-plate agar method.

For pH stability, phages with a titer of 10^10^ PFU/ml were mixed in a series of different SM buffers (pH 2–11, adjusted using NaOH or HCl) at 30°C, and samples were taken at 1 h, 2 h, and 3 h to determine the titers by the double-plate agar method.

For UV stability, 4 mL of phage sample with a titer of 10^10^ PFU/ml was spread on a sterilized petri dish (d = 90 mm) and placed on a bechtop (SX-BHC-1300A2, Suzhou Suxin Environmental Technology Co., Ltd.) at a vertical distance of 40 cm under UV light (400 mW/m^2^,). The samples were taken for titer determination every 10 min.

For one-step growth curve, 5 mL of phage (1 × 10^8^ PFU/ml) was mixed with 5 mL of fresh host bacterial (NdE strain OD_600_ = 0.863 was about 1.73 × 10^9^ CFU/mL, dilution with sterile water) at an MOI of 1 and incubated at 30°C for 5 min with 180 rpm. Then, the samples were centrifuged at 10,000 g for 5 min at 4°C, and the pellet was re-suspended with NB; this process was repeated three times. The final suspension was added to 10 mL of NB and cultured at 30°C, 180 rpm. Phage titers were measured at 0 min, 15 min, and 30 min and followed 30 min intervals in triplicate using the double-layer agar method. The burst size was calculated by dividing the final phage titer by the initial phage titer.

All tests were repeated three times.

### 2.5 Sequencing and bioinformatic analysis

Genomic DNA of the phage was extracted using the Norgen Biotek Phage DNA Isolation Kit (Norgen Biotek, Canada). The concentration and purity of isolated genomic DNA was assessed according to the protocol of the kit. DNA samples were subjected to paired-end sequencing on the Illumina HiSeq sequencing platform. The short reads were assembled by SOAPdenovo (version: 2.04) and GapCloser (version: 1.12) to obtain the whole genome of phage. Phage ORFs were analyzed using GeneMarks (Besemer and Borodovsky, 2005), and functional protein-coding genes were predicted by searching against the National Center for Biotechnology lnformation Database using Diamond software (Buchfink et al., 2015). Virulence genes and antibiotic resistance genes were searched for in the VFDB (Chen et al., 2005) and CARD (McArthur et al., 2013) databases. A circle map of the phage genome was generated with CG View (Stothard and Wishart, 2005). The genome comparisons between phages were performed with Easyfig 2.2.5 (Sullivan et al., 2011). Bioinformatics tool vContact3 (v 0.0b39; database v220; accessed on May 9, 2024; https://bitbucket.org/MAVERICLab/vcontact3/src/master/) and VICTOR (Virus Classification and Tree Building Online Resource with the amino acid option selected, Available online: https://ggdc.dsmz.de/victor.php#) (accessed on May 10, 2024) were used for phage taxonomic classification (Gendre et al., 2022; Zhu et al., 2022). Intergenomic nucleotide sequence similarity were aligned and plotted by VIRIDIC with recommended configurations (Moraru et al., 2020). Phage genome-wide nucleic acid sequence and three “signature gene products” (tail fiber protein, portal protein, and terminase large subunit protein) of amino acid sequences were used for phylogenetic analysis by MEGA with Maximum Likelihood method (Kumar et al., 2018).

### 2.6 Assessment of PQ43W activity against tobacco BW

#### 2.6.1 Pot experiment

A pot experiment was conducted to evaluate the efficacy of bacteriophage against tobacco BW disease and to determine the optimal dose. The pot experiment was carried out according to the method described by Le et al. (2020) and Eisfeld et al. (2022) with slight modifications. Tobacco seedlings were transplanted to a pot at the four-leaf stage, one seedling was used per pot and 500 g nursery substrate was added (Xiang Hui Agriculture Co., Ltd., China). After seedlings rooted, the experiments were grouped and performed based on Table 1; each group had three replications (18 seedlings per replication). BW pathogen *R. ps.* NdE (10^5^ CFU/mL * 20 mL per seedling) was inoculated with BW by non-invasive root irrigation. After the pathogen inoculated for 6 h and 72 h, the phage agents (10^8^ PFU/ml) with different dilutions (0, 10, 50, 100, and 200) were used with the same method (non-invasive root irrigation, 20 mL per seedling) followed by cultivation in a suitable climate. The temperature was 28 ± 3°C, humidity 60%–90%, 14 h light-10 h dark, and full spectrum 6,000 lm. Sterile water (about 25 mL/pot) was added to the pot every 2–5 days to maintain the humidity of the nursery substrate in pot at 25%–40%.

**Table 1:** Pot experimental groups.

Groups	Experimental treatments	
T1	*R. ps.* NdE + phage stock solution	Non-invasive root irrigation (20 mL -times / seedings); phage inoculation at 6 h and 72 h after *R. ps.* NdE inoculation.
T10	*R. ps.* NdE + phage 10-fold dilution	
T50	*R. ps.* NdE + phage 50-fold dilution	
T100	*R. ps.* NdE + phage 100-fold dilution	
T200	*R. ps.* NdE + phage 200-fold dilution	
CK+	*R. ps.* NdE + sterile water	
CK-	Equal amounts of sterile water	

After 30 days of pathogen inoculation, the BW incidence rate, disease index, and relative control effect of phage agents were calculated according to the GB/T23222-2008 (Grade and investigation method of tobacco diseases and insect pests) (National public service platform for standards information, n.d.). The tobacco plant BW disease classification level was assessed on a scale of 0–9: 0 = no symptoms; 1 = half of the leaves on the diseased side were wilted or withered; 3 = black strip on stem, but not more than half of the stem height, or half to two-thirds of the diseased side leaves withered; 5 = the black strip on the stem exceeds half of the stem height, but does not reach the top of the stem, or more than two thirds of the leaves on the diseased side are wilted; 7 = the black spot on the stem reaches the top of the stem, or the leaves of the diseased plant are all withered; and 9 = a dead plant.

The BW incidence rate (IR) was the BW disease plants ratio; the disease index (DI) and control effect (CE) were calculated with Eqs 1, 2.

The calculation formula of DI and CE is as follows:


(1) $$DI=100*\left(1*n1+3*n2+5*n3+7*n4+9*n5\right)/\left(n*9\right)$$

(2) $$CE=\left(C-T\right)/C$$

DI: disease index; n1–n5: the number of plants in each disease classification level; n: the number of plants investigated; CE: control effect (T versus C); C: disease index of control group; T: disease index of treatment group.

#### 2.6.2 Field experiment

The experimental field is located in a tobacco field (28°13’ N, 112°32′ E) in Yuxin Village, Shuangfu Town, Ningxiang City, Hunan Province, China. The area has a subtropical humid monsoon climate; in the tobacco growing season (March to July), the average monthly temperature and average monthly precipitation were 21.6°C and 168 mm. The experimental site was 56 m above sea level, and the soil was brown soil with a pH value of 6.13, organic matter 17.84 g/kg, total nitrogen 1.29 g/kg, alkaline nitrogen 83.14 mg/kg, available phosphorus 113.91 mg/kg, and available potassium 254.36 mg/kg. The tobacco cultivar Yunyan 87 was tested. The tobacco seedlings were transplanted at the five-leaf seedling stage on March 20. The experiment was divided into three groups, namely the control group (FCK) without treatment, the General fungicide group (FZS), where a chemical fungicide – Zhongshengmycin (Yi et al., 2022) – was applied according to the recommended dosage, and the Phage treatment group (FPQ43W), where phage was used as 10^8^ PFU/ml-20 mL-plants. All the agents were applied twice by non-invasive root irrigation on 20 May and 20 June. The disease survey was carried out on July 10 (about a week that before the tail leaves were harvested) according to the method described in the pot experiment above.

### 2.7 Statistical analysis

The test data were processed by Excel; SPSS software was used for statistical analysis. Duncan’s method of new repolarization (DMRT) was used to test the significant differences.

## 3 Results

### 3.1 Isolation and morphological characterization of a lytic bacteriophage

#### 3.1.1 Isolation and characterization of a bacterial wilt pathogen

**Figure 1:**
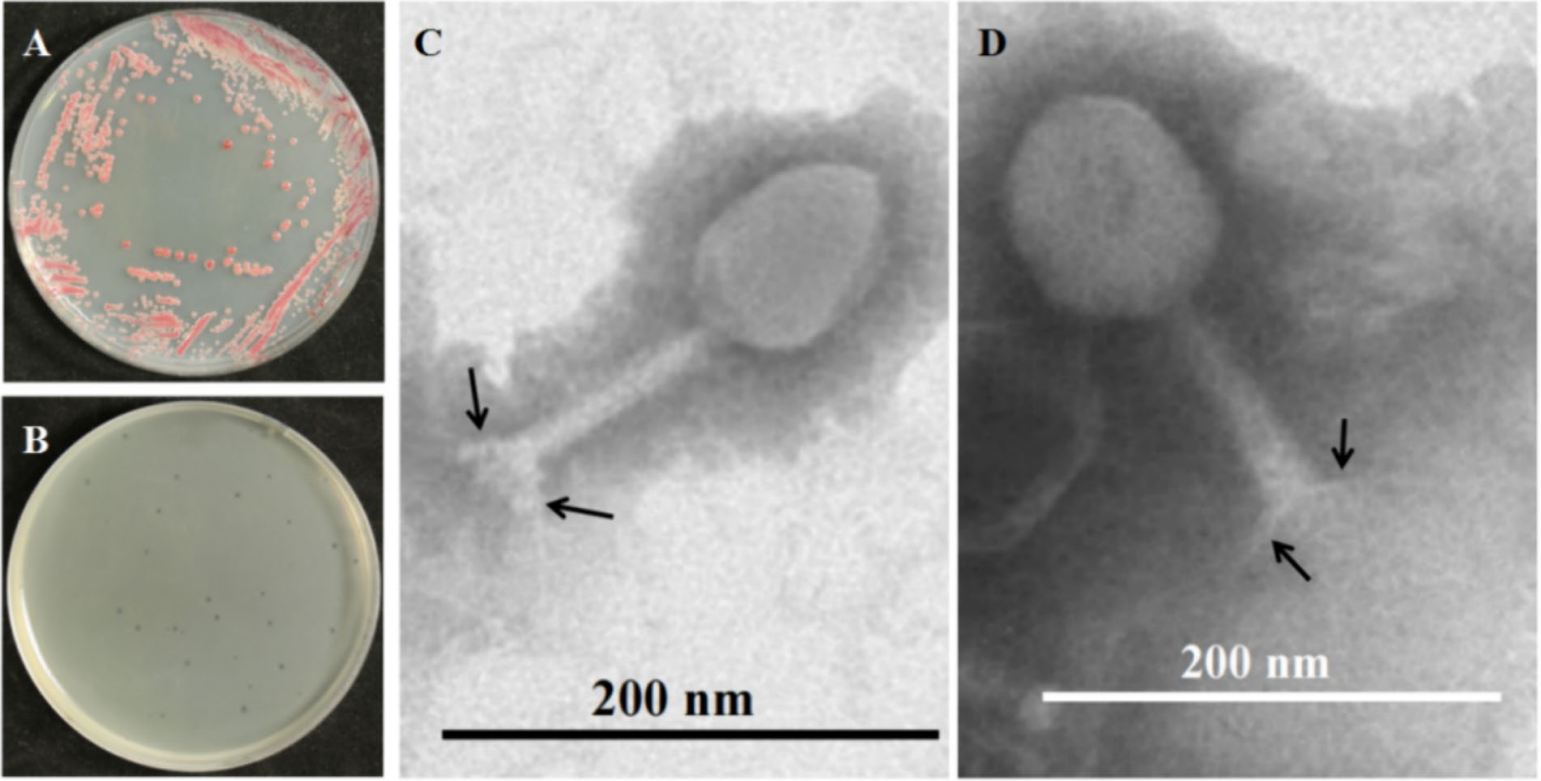
Morphology of BW pathogen *Ralstonia pseudosolanacearum* NdE (*R. ps.* NdE) and its phage PQ43W. **(A)** Colony morphology of *R. ps.* NdE on TTC plate; **(B)** formed clear plaques on a lawn of *R. ps.* NdE double-plates; **(C,D)** Virions displaying typical polygonal heads, long tails, and tail fibers (arrows) of the Class *Caudoviricetes* by TEM.

The bacteria, as shown in Figure 1A, had red smooth colonies on TTC plates. The bacteria were isolated from tobacco BW disease stem with bacterial ooze by plate streaking in Hunan, China, and named NdE. A blastn analysis in NCBI showed that strain NdE had >99% similarity with the bacteria *Ralstonia solanacearum* species complex (Rssc) (Supplementary Table S1). A phylogenetic analysis showed that strain NdE was clustered into a cluster with *Ralstonia pseudosolanacearum* (NR_134148.1) and placed into Rssc clade (Supplementary Figure S1). Therefore, the bacteria NdE identified as *Ralstonia pseudosolanacearum* is a member of Rssc.

#### 3.1.2 Isolation and characterization of a lytic bacteriophage

The phage was isolated from rhizosphere soil, which was collected from a field in Hunan Province, China with a high incidence of tobacco BW. The phage produced small and clear plaques with a diameter of approximately 1–2 mm after overnight incubation at 28°C on the top layer containing 0.75% agar of a double-layered NB plate using *R. ps.* NdE strain as a host (Figure 1B); the phage was named *Ralstonia* phage PQ43W. Transmission electron microscopy (TEM) was used to visualize the morphology and for classification (Figures 1C,D). The phage has a polygonal head with an average capsid diameter of 86 ± 4 nm (*n* = 10), a long tail with a length of 123 ± 3 nm and a width of 18 nm ± 3 nm (*n* = 10), and a baseplate and tail fibers of approximately 20 ± 2 nm (*n* = 10) in length (Figure 1B). Based on the morphology and structural features of the phage virions, it is typical for members of class *Caudoviricetes*, according to the standard of the International Committee on Taxonomy of Viruses (ICTV).

### 3.2 Biological characteristics

**Figure 2:**
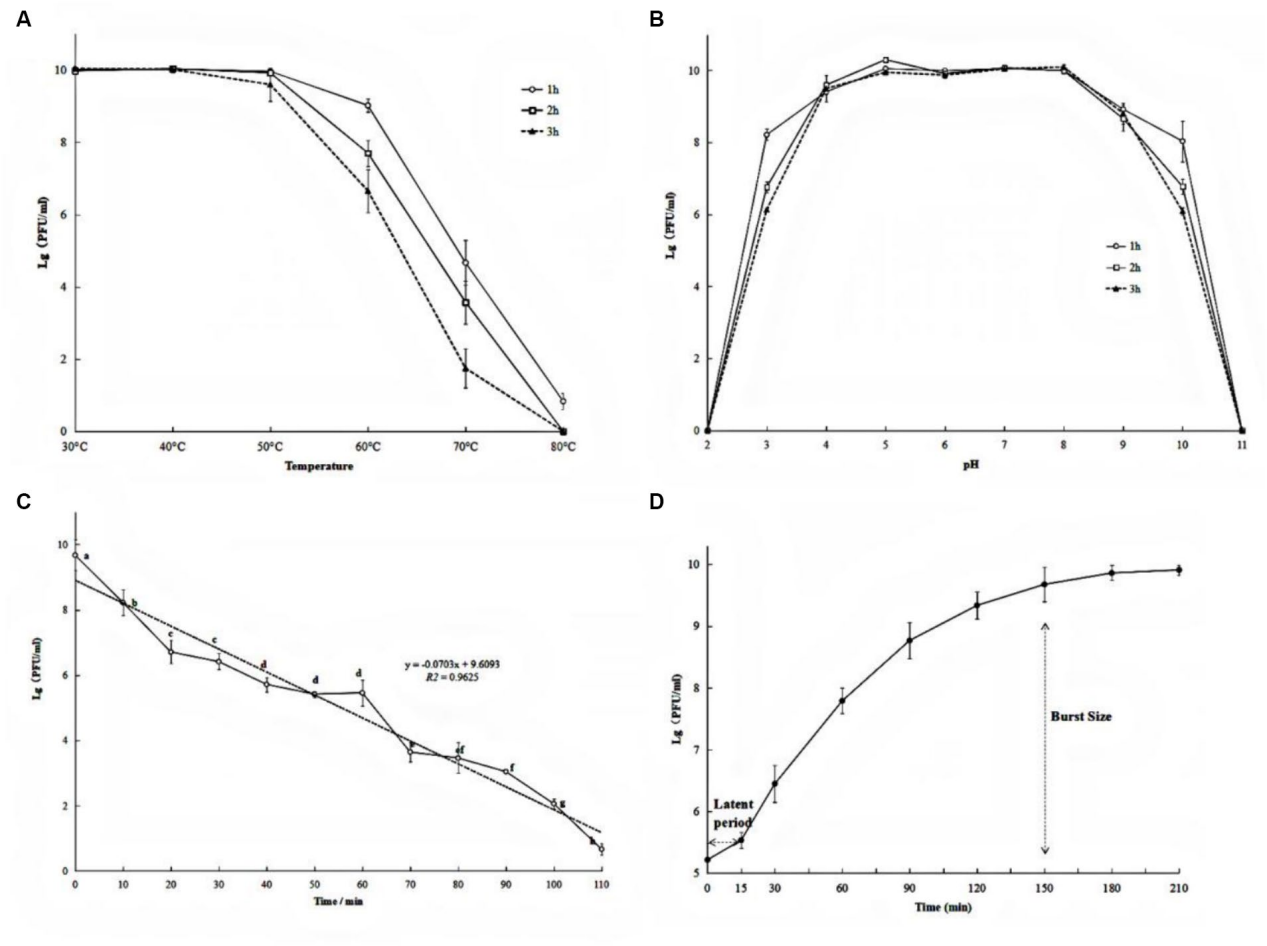
Biological characteristics of phage PQ43W on thermal stability **(A)**, pH **(B)**, UV **(C)** tolerance, and one-step growth curve **(D)**. Values are the mean of three determinations.

The exploitation of the phage as an antibacterial agent requires a wide range of environmental adaptations including tolerance to high temperatures, acid-alkaline environments, and ultraviolet radiation. After incubation at 30°C, 40°C, and 50°C, no significant change in the phage number was observed (Figure 2A). However, after incubation at 60°C for 1 h, 2 h, and 3 h, the phage titer decreased by 0.96 log, 2.21 log, and 3.15 log, respectively (Figure 2A). A remarkable decrease in phage titer after incubation at 70°C was observed (4.87, 3.78, and 1.94 units log after 1 h, 2 h, and 3 h, respectively). Similarly, the phage titer fell below 1 log when treatment was carried out at 80°C (*p* < 0.05). These results suggest that phage PQ43W is only stable at temperatures <50°C.

With respect to pH sensitivity, phage PQ43W was fairly stable in the range of pH 4 to 9 (Figure 2B). In particular, the titer changed less than 0.5 log units at pH 5–8, regardless of whether the incubation time was 1 h, 2 h, or 3 h. However, at pH 3 and 10, the phage titer decreased sharply, decreasing by 2.02 to 3.95 and 1.84 to 3.91 unit log, respectively, when the incubation time was 1–3 h. This indicated that phage PQ43W has a broad pH tolerance, especially in the range of pH 5 to 8.

The results of UV tolerance experiments demonstrated that the phage titer decreased sharply after exposure to UV irradiation (Figure 2C). After 60 min of UV irradiation, the titer of phage PQ43W decreased by about half the log units to only 5.46 unit log. As the UV exposure time increased to 110 min, the phage titer fell to 1 log.

To further understand the life cycle and infection kinetics of PQ43W, a one-step growth curve using a MOI of 1 to infect host *R. ps.* NdE cultures was examined and shown in Figure 2D. The results showed (Figure 2D) a latent period of 15 min and a rapid growing period of 15–150 min, with burst sizes of 74.20 ± 18.88 PFU/infected cell at periods of 150 min.

### 3.3 Genomic analysis

#### 3.3.1 Genomic features

**Figure 3:**
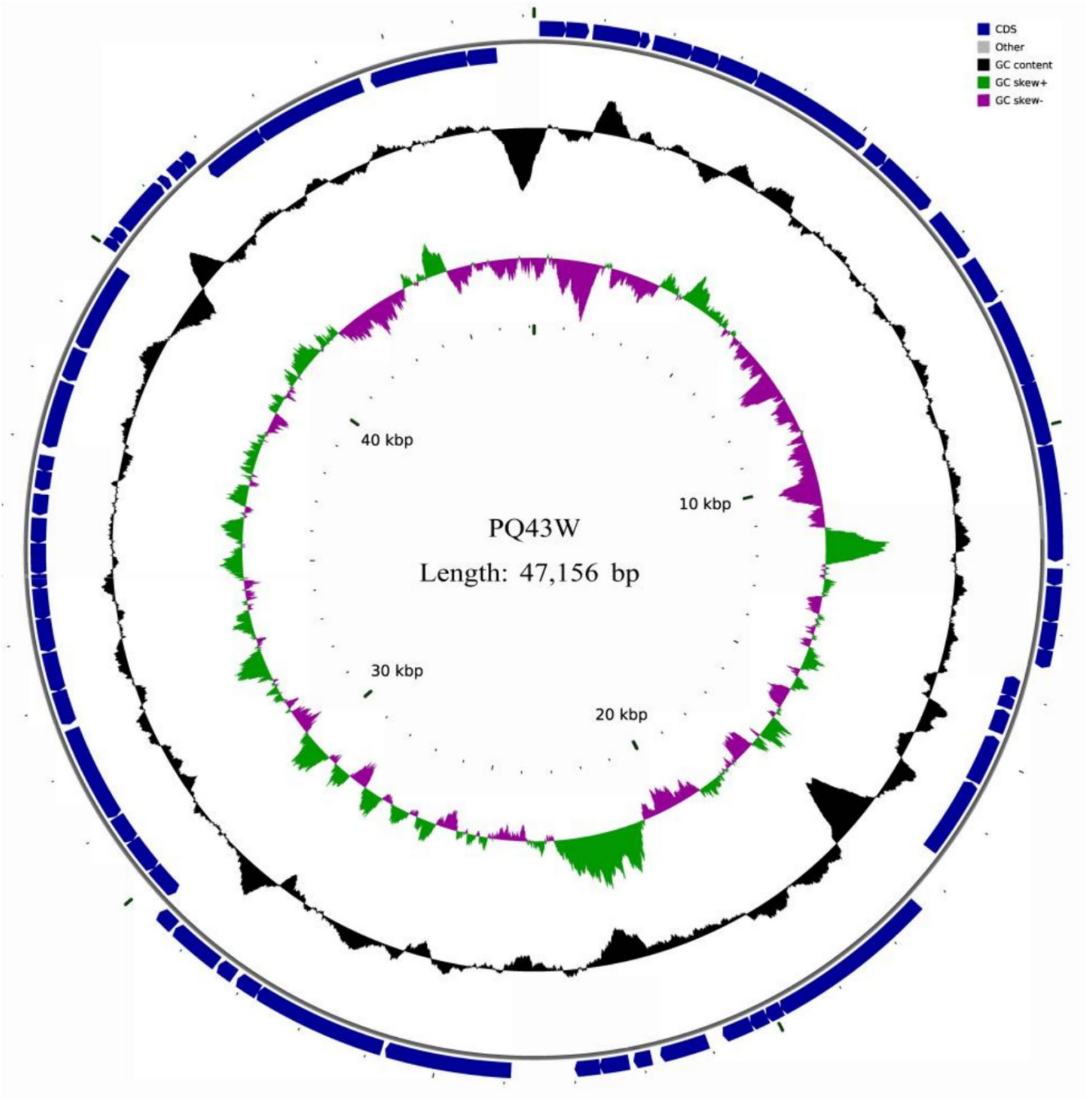
Circle map of phage PQ43W genome. GC skew and GC content are shown in the inner circle. ORFs are visualized in the outer circle with arrows indicating the transcription direction.

Genome sequencing showed that the phage PQ43W genome was dsDNA and circular, and the complete genomic DNA sequence (GenBank: PP405626) indicated that its genome was 47,156 bp long with guanine-cytosine content of 64.27%. The contents of G, C, A, and T were 32.45, 31.82, 17.73, and 18.00%, respectively. It has 65 predicted open reading frames (ORF1–ORF65); 39 ORFs were encoded on the plus strand and 26 ORFs were encoded on the minus strand (Figure 3; Supplementary Table S2). The total length of the ORFs sequence was 42,516 bp and the average length was 654 bp per ORFs. The coding percentage was 90.16%.

#### 3.3.2 Gene annotation of predicted proteins

A total of 65 potential open reading frames (ORFs) were identified (Figure 3; Supplementary Table S2). Of them, seven had no significant similarity with any of the protein sequences in the searched databases and 36 were annotated as conserved, conserved hypothetical, or unnamed proteins with no assigned functions. Only 29 were predicted proteins that shared homology with other phages or bacteria with assigned functions based on KEGG analysis (Table 2). Of them, there were six phage-associated DNA replication and repair proteins, including RusA-like Holliday junction resolvase (ORF22), exonuclease (ORF24), DNA primase (ORF25), DNA helicase (ORF33), DNA polymerase (ORF34), and adenosine (5′)-pentaphospho-(5″)-adenosine pyrophosphohydrolase (ORF40). There were two phage-associated lysis proteins: holin (ORF16) and lysozyme (ORF17). There was one phage-associated packaging gene: terminase large subunit (ORF64). There was one phage-associated transcriptional regulator: phelix-turn-helix transcriptional regulator (ORF26). There were 19 phage-associated structural genes proteins, such as baseplate wedge subunit (ORF1) and virion structural protein (ORF2).

**Table 2:** List of annotated ORFs of phage PQ43W with predicted functions and proteins and their BLASTp results.

Function	ORFs	Strand	Start (nt)	End (nt)	Len. of protein (aa)	Amino acid sequence identity/ similarity to homologs	Homologs		
							E-value	Identity	Accession No.
Structural genes	ORF1	+	77	466	129	Baseplate wedge subunit [*Ralstonia* phage Adzire]	5e-39	55.65%	YP_010052725.1
	ORF2	+	463	795	110	Virion structural protein [*Ralstonia* phage Adzire]	5e-33	58.00%	YP_010052726.1
	ORF6	+	2,310	2,714	134	Tail assembly chaperone [*Burkholderia* phage Bcep781]	2e-29	44.36%	NP_705670.1
	ORF8	+	3,322	5,142	606	Tail length tape measure protein [*Xanthomonas* phage NEB7]	4e-139	46.44%	WHB31172.1
	ORF10	+	5,522	6,418	298	Baseplate hub [*Xanthomonas* phage KPhi1]	1e-109	51.55%	YP_010052608.1
	ORF11	+	6,578	7,309	243	baseplate assembly protein [*Burkholderia* phage BcepNY3]	1e-79	53.14%	YP_001294874.1
	ORF14	+	9,357	10,277	306	Structural protein [*Xanthomonas* phage KPhi1]	4e-64	69.50%	YP_010052604.1
	ORF15	+	10,287	11,960	557	Tail fiber protein [*Burkholderia* phage Bcep1]	7e-83	57.14%	NP_944342.1
	ORF31	+	22,204	22,632	142	Major tail protein with Ig-like domain [*Ralstonia* phage AhaGv]	3e-10	47.13%	WRQ05499.1
	ORF41	−	30,660	31,091	143	Virion structural protein [*Burkholderia* phage Bcep781]	1e-56	65.73%	NP_705630.1
	ORF45	−	33,812	34,315	167	Head protein [*Burkholderia* phage Bcep43]	1e-54	59.26%	NP_958110.1
	ORF46	−	34,312	34,779	155	Tail completion or Neck1 protein [*Xanthomonas* phage KPhi1]	1e-48	54.84%	YP_010052575.1
	ORF48	−	34,997	35,464	155	Virion structural protein [*Burkholderia* phage Bcep1]	4e-65	65.79%	NP_944316.1
	ORF53	−	36,925	37,944	339	Major head protein [*Ralstonia* phage Adzire]	0	71.98%	YP_010052715.1
	ORF54	−	37,979	38,479	166	Minor head protein [*Burkholderia* phage Bcep781]	7e-84	75.15%	NP_705639.1
	ORF55	−	38,516	39,838	440	Head maturation protease [*Burkholderia* phage Bcep781]	1e-122	49.47%	NP_705640.2
	ORF62	−	41,824	42,777	317	Head morphogenesis [*Burkholderia* phage Bcep781]	1e-103	55.75%	NP_705642.1
DNA replication and repair	ORF22	−	14,251	14,610	119	RusA-like Holliday junction resolvase [*Xanthomonas* virus phiXaf18]	7e-42	63.25%	YP_010052683.1
	ORF24	−	15,458	16,654	398	Exonuclease [*Burkholderia* phage Bcep1]	1e-125	52.84%	NP_944329.1
	ORF25	+	17,393	19,876	827	DNA primase [*Burkholderia* phage Bcep781]	0	59.39%	NP_705675.1
	ORF33	+	23,910	25,751	613	DNA helicase [*Burkholderia* phage Bcep43]	0	74.80%	NP_958163.1
	ORF34	+	25,802	27,766	654	DNA polymerase [*Burkholderia* phage Bcep43]	0.0	68.75%	NP_958166.1
Lysis	ORF16	+	12,062	12,307	81	Holin [*Curvibacter* phage P26059B]	4e-11	50.00%	YP_009811766.1
	ORF17	+	12,318	12,836	172	Lysozyme [Paraburkholderia caballeronis]	2e-49	51.92%	WP_167627125.1
Transcriptional regulator	ORF26	+	19,873	20,112	79	helix-turn-helix transcriptional regulator [*Eubacterium* sp.]	7e-08	47.69%	MCI9022338.1
Packaging genes	ORF64	−	44,639	46,129	496	Terminase large subunit [*Burkholderia* phage Bcep781]	0.0	70.33%	NP_705644.1

#### 3.3.3 Comparison genome

**Figure 4:**
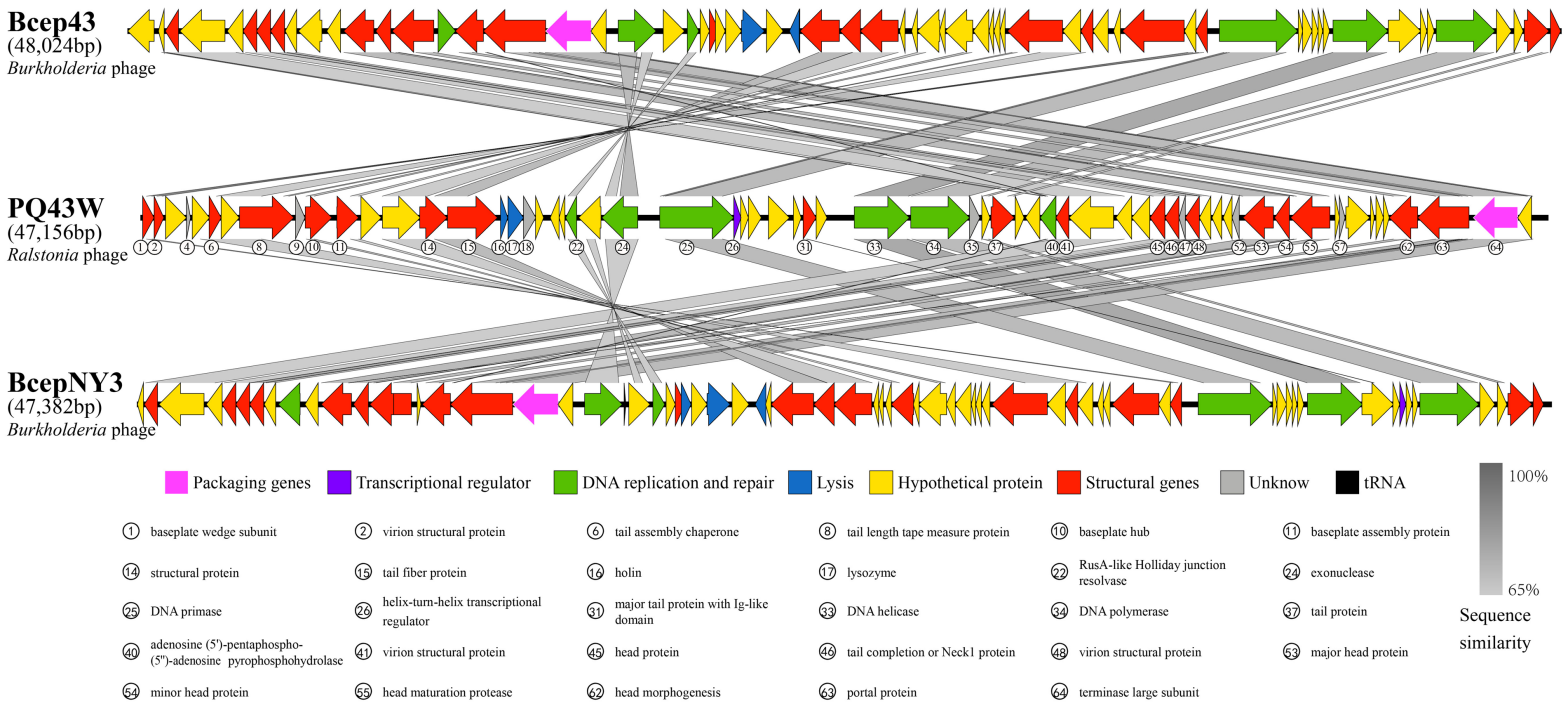
Comparison of the complete genome of PQ43W and two related phages, *Burkholderia* phage Bcep43 (Supplementary Table S3) and *Burkholderia* phage BcepNY3 (Supplementary Table S4). Gray bands show similarity in nucleotide sequences, arrows represent open reading frames, and the different colors of ORFs indicate the functional classification of the genes.

Figure 4 shows a blastn search using the nucleotide in NCBI Genbank that was homologous to the *Burkholderia* phage Bcep43 (NC_005342.2, 16% query coverage, and 76.55% identity) and *Burkholderia* phage BcepNY3 (NC_009604.1, 9% query coverage and 76.52% identity) with the highest score. It also shows the genome comparison analysis of Bcep43 and BcepNY3. The gene arrangement structure of PQ43W was significantly different from that of Bcep43 (Supplementary Table S3) and BcepNY3 (Supplementary Table S4). However, there were some similarities in some regional sequences (such as ORF1 ~ ORF15; ORF22 ~ ORF24; ORF25; ORF33 ~ ORF36; ORF40 ~ ORF65). Compared with Bcep43 or BcepNY3, the gene arrangement structure is very different, and the genes with the same function had different distribution positions and directions in the genome.

#### 3.3.4 Taxonomic and phylogenetic analysis

As further taxonomic information was not confirmed by the morphological identification, we resorted to genome-wide analyses at the protein level to clarify further taxonomic classification information with vContact3. The results in Table 3 show that PQ43W belongs to a novel genus of subfamily *Kantovirinae* (Table 3; Supplementary Table S5).

**Table 3:** Prediction result of phage PQ43W taxonomic classification by vContact3.

GenomeName	PQ43W
Realm (prediction)	*Duplodnaviria*
Phylum (prediction)	*Uroviricota*
Class (prediction)	*Caudoviricetes*
Order (prediction)	novel_Order_17_of_*Caudoviricetes*
Family (prediction)	novel_Family_0_of_novel_order_17_of_*Caudoviricetes*
SubFamily (prediction)	*Kantovirinae*
Genus (prediction)	novel_Genus_0_of_*Kantovirinae*

In addition, the classification with VICTOR using the “amino acid” option analysis to build phylogenetic trees was refined and constructed. Since the reference sequence of phylogenetic trees is directly related to the analysis result, it is very important to select appropriate, taxonomically close and representative sequences. So, in this study the reference sequence for VICTOR analysis were from five parts: (i) all the phages in subfamily *Kantovirinae* classification in ICTV Taxonomy Browser (https://ictv.global/taxonomy; two genera, five species, five phages); (ii) all the phages at subfamily *Kantovirinae* classification in NCBI taxonomy browser (https://www.ncbi.nlm.nih.gov/Taxonomy/Browser/wwwtax.cgi?id=2946634; two genera, five species, 13 of no rank, 18 phages); (iii) all the phages at subfamily *Kantovirinae* (Prediction) classification by vContact3 (database v220) (Supplementary Table S5; four genera, 12 phages.); (iv) Top 12 phages with the highest similarity in NCBI blastn with PQ43W genome-wide sequence (Supplementary Table S6; three genera, 12 phages); (iv) two *Ralstonia* phages were not in the subfamily *Kantovirinae* classification in ICTV and NCBI taxonomy database (Supplementary Table S6, Genus *Cimandefvirus*) and were used as outgroups. Subsequently, 32 phages were selected and listed in Supplementary Table S7 for further analysis.

The VICTOR analysis of phylogenomic GBDP trees inferred with the formulas D0, D4, and D6 were shown in Supplementary Figures S2–S4, respectively. Then, a summarized result based on the prediction taxonomy was shown in Supplementary Table S8. The prediction taxonomic of PQ43W showed the same results at the Phylum, Subfamily, and Genus classifications in formulas D0 and D6. PQ43W belongs to a novel Subfamily and novel Genus that does not have the same taxonomic phage at Subfamily and Genus taxonomic classification levels. In the formula of D4, the prediction taxonomic showed PQ43W with *Ralstonia* phage Eline (NC 054957.1) and *Ralstonia* phage Gerry (NC 054959.1) has the same taxonomic classification at Genus *Cimandefvirus* (Family: unknown; Subfamily: unknown). Considering the yielding average supports were 73, 69, and 76% at the formulas D0, D4, and D6, respectively, the prediction taxonomic results of PQ43W inferred in formulas D0 and D6 were more accurate than D4. In addition, eight phage predictions in Subfamily *Kantovirinae in* vContact3 also appeared in the prediction Family classification through VICTOR analysis with formulas D0 and D6, but the results analyzed with formula D4 was not. That also indicated the formulas D0 and D6 were more suitable for PQ43W by VICTOR analysis than the formula D4. So, PQ43W may belong to a novel Genus in Subfamily *Kantovirinae.*

**Figure 5:**
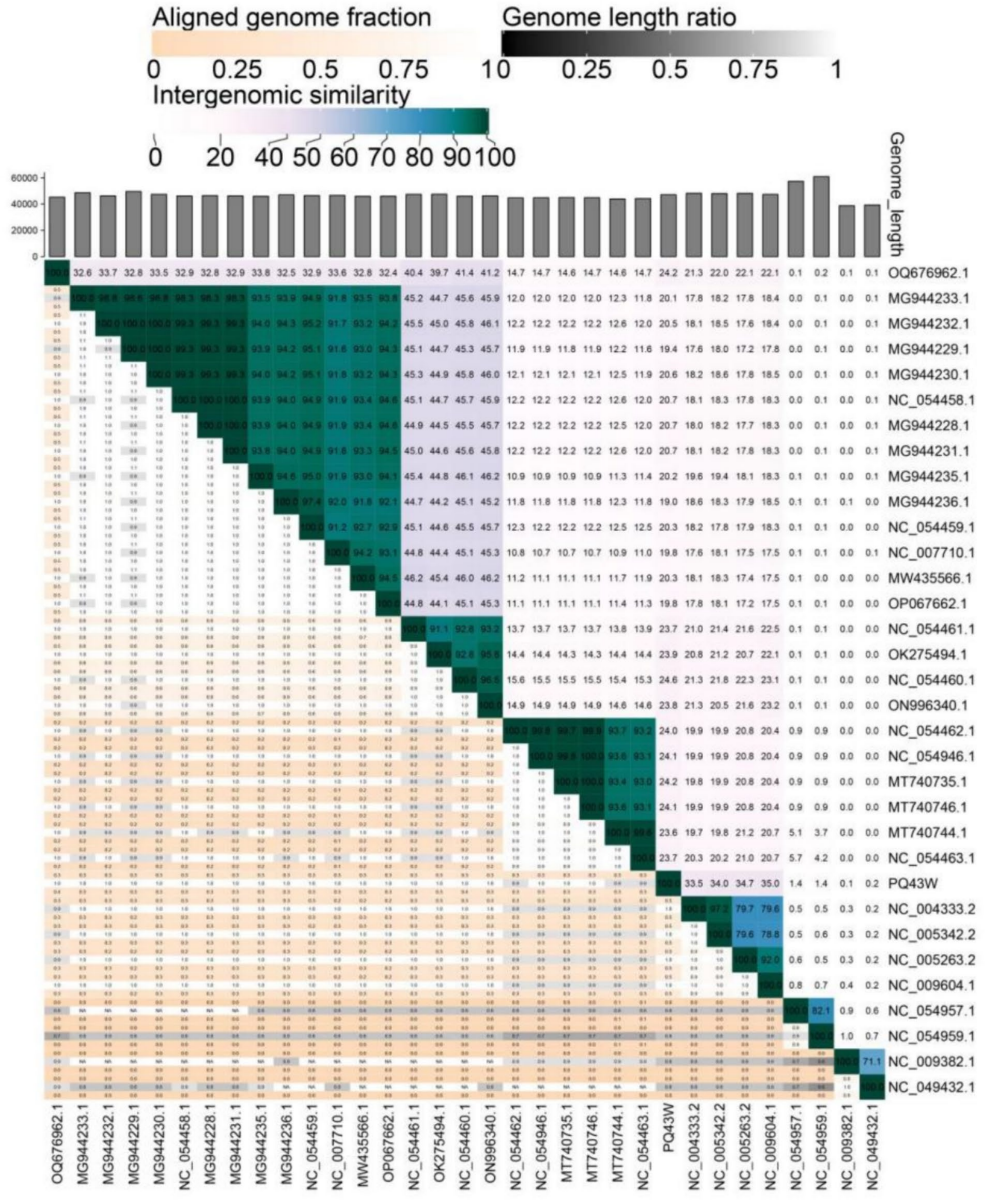
Intergenomic similarity between PQ43W and its 32 related phages, calculated using VIRIDIC. The right half of this heatmap shows the similarity values between phage genomes. The left half of this heatmap shows the aligned genome fraction and genome length ratio.

A comparative analysis of genomic nucleotide similarity by VIRDIC was shown in Figure 5 and Supplementary Table S8 with 32 other related phages (Supplementary Table S7). The results of intergenomic similarity between PQ43W and these phages were 0.1–35.0% (Figure 5), and the species and genus clusters table is different from the 32 related phages (Supplementary Table S9). Based on the principle of classifying bacteriophage on genus in Bacterial and Archaeal Viruses Subcommittee (BAVS), a genus is a cohesive group of viruses sharing a high degree of nucleotide sequence similarity (>50%) that is distinct from viruses of other genera (Adriaenssens and Brister, 2017). So, PQ43W belongs to a novel genus in the class *Caudoviricetes.*

Therefore, PQ43W belongs to a novel genus of subfamily *Kantovirinae* under class *Caudoviricetes*.

**Figure 6:**
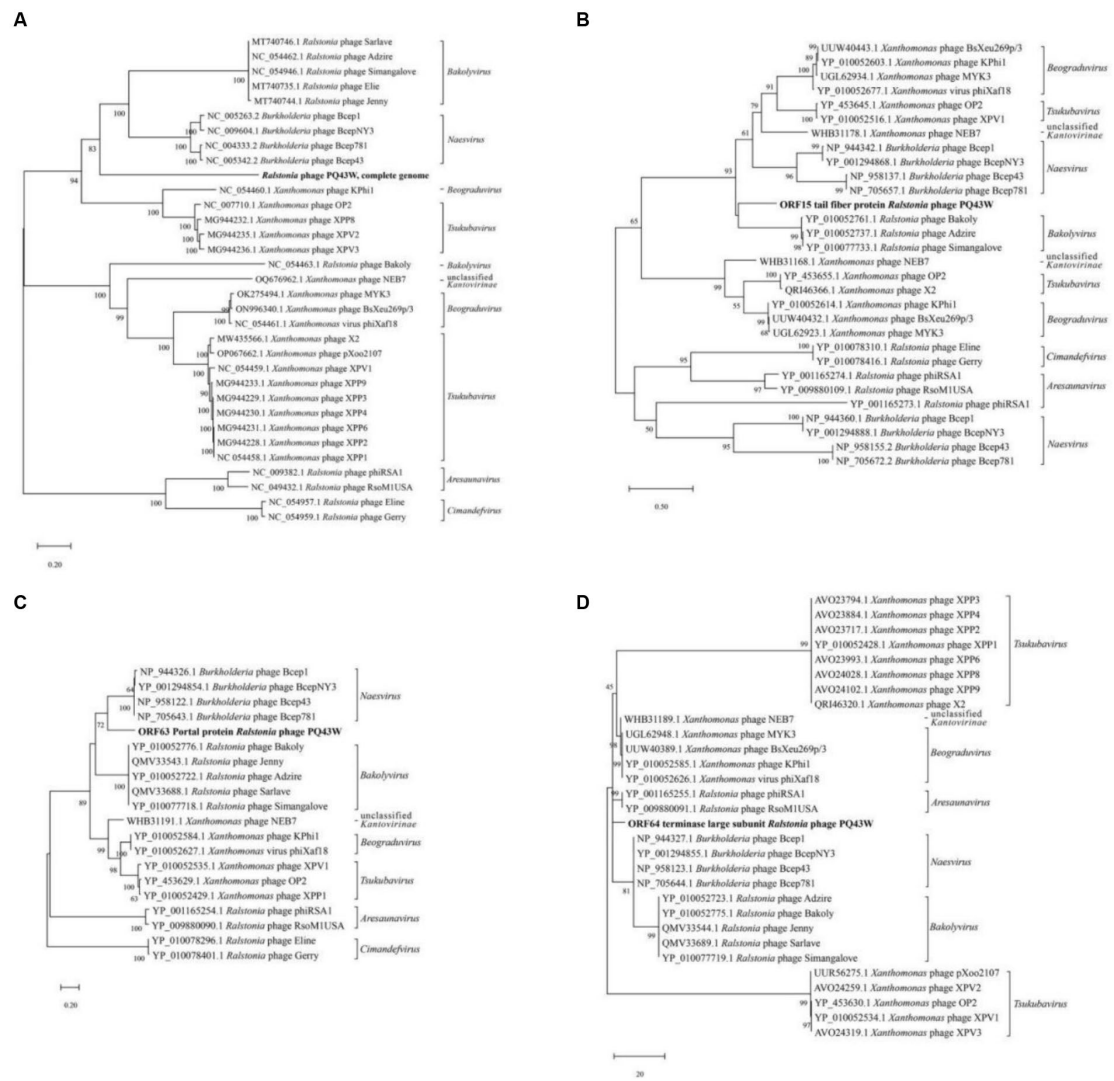
The phylogenetic trees were constructed using maximum likelihood (ML) method by MEGA, based on the genome-wide nucleic acid sequence **(A)** and three “signature gene products” that annotated tail fiber protein **(B)**, portal protein **(C)**, and terminase large subunit protein **(D)**. Vertical distances are arbitrary, but the horizontal branches are proportional to genetic distance. Bootstrap values (1,000 replications) are represented at the nodes of the branches. Taxonomic classification of genera were labeled in the right of phylogenetic trees. The cluster of PQ43W was shown in bold.

To determine evolutional relationships between PQ43W and its 32 related phages (Supplementary Table S7), a phylogenetic tree based on the genome-wide nucleic acid sequence showed that PQ43 is adjacent to the genera of *Naesvirus, Bakolyvirus, Beograduvirus*, and *Tsukubavirus*, but distinct from them, in a separate clade (Figure 6A). In addition, the phylogenetic tree based on the three “signature gene products (tail fiber protein, portal protein, and terminase large subunit)” (Addy et al., 2019; Khan et al., 2021; Sasaki et al., 2021) of amino acid sequences showed that PQ43W is more closely related to the clade genera of *Bakolyvirus* (Figure 6B), *Naesvirus* (Figure 6C), or *Aresaunavirus* (Figure 6D), but is not in their clades. So, PQ43W has closer evolutional relationships to the genera *Naesvirus, Bakolyvirus, Beograduvirus, Tsukubavirus*, *or Aresaunavirus* in the phylogenetic analysis, though PQ43W belongs to a novel genus and was placed in a clade alone in Figure 6.

### 3.4 Phage therapy

#### 3.4.1 Pot experiment

A pot experiment to address phage application for the control of tobacco BW disease caused by *R. ps.* NdE was conducted. An obvious control effect result on tobacco BW disease was surveyed and statistically analyzed after 30 days (Supplementary Figure S6; Table 4). It is easily observed in Supplementary Figure S6 that PQ43W could decrease the tobacco BW disease incidence rate (IR) and disease index (DI), and was consistent with the disease statistics result (Table 4). The PQ43W application groups showed a lower BW incidence rate (IR) and disease index (DI) to compared with the control group (CK+) in Table 4. On the whole, a higher titer of PQ43W application groups showed a better control effect on tobacco BW that the IR and DI indices were lower, especially the groups T1 and T10 had more than 50% CE on the control of tobacco BW for 30 days. What’s importantly, group T1 of the CE index increased to 92.80% for 30 days by root irrigation application at 10^8^ PFU/ml titers significantly.

**Table 4:** Effect of PQ43W on controlling tobacco bacterial wilt caused by *R. ps.* NdE.

Groups	Treatments	Incidence Rate (IR) %	Disease Index (DI)	Control Effect (CE) %
T1	*R. ps.* NdE + PQ43W	10.08 ± 3.34 d	6.17 ± 6.72 c	92.80 ± 6.83 a
T10	*R. ps.* NdE + PQ43W (10×)	42.22 ± 20.37 bcd	35.31 ± 19.56 bc	58.84 ± 22.80 b
T50	*R. ps.* NdE + PQ43W (50×)	73.33 ± 13.33 abc	48.89 ± 4.64 abc	43.02 ± 5.41 bc
T100	*R. ps.* NdE + PQ43W (100×)	68.67 ± 5.03 abc	51.11 ± 13.02 abc	40.43 ± 15.18 bc
T200	*R. ps.* NdE + PQ43W (200×)	80.00 ± 6.67 ab	65.43 ± 23.12 ab	26.74 ± 13.95 c
CK+	*R. ps.* NdE	90.67 ± 3.53 a	87.80 ± 3.02 a	0.00 ± 0.04 d
CK-	–	0.00 ± 0.00 e	0.00 ± 0.00 d	–

The data in the table are the results after 30 days of counting. Values are means ± standard deviation, and letters following values indicate only the level of Duncan’s analysis for values in the same column (*p* < 0.05).

#### 3.4.2 Field experiment

Subsequently, a field experiment was conducted and the result showed in Table 5, based on the optimal dose of pot experiment (Table 4; T1: 10^8^ PFU/ml). Compared to the control treatment (FCK), PQ43W treatment (FPQ43W) had a lower Incidence Rate (IR) that was significantly reduced from 69.33% to 30.00% (*p* < 0.05). The Disease Index (DI) was also significantly lower and reduced from 17.19 to 3.78% (*p* < 0.05). In addition, the Control Effect (CE) of PQ43W treatment (FPQ43W: 78.02%) was significantly better than the traditional pesticide Zhongshengmycin treatment (FZS: 44.41%), (*p* < 0.05). At the same, the indexes of IR and DI in FPQ43W were lower than FZS, significantly (*p* < 0.05). Therefore, PQ43W of phage therapy to control BW in pot and field experiments indicated that PQ43W is a promising resource in controlling tobacco BW disease.

**Table 5:** Effects of different treatments on controlling the tobacco bacterial wilt in field experiment.

Treatments	Incidence rate % (IR)	Disease index (DI)	Control effect % (CE)
FCK	69.33 ± 4.62 a	17.19 ± 1.03 a	–
FZS	48.67 ± 5.03 b	9.56 ± 1.98 b	44.41 ± 11.49 a
FPQ43W	30.00 ± 7.21 c	3.78 ± 1.56 c	78.02 ± 9.05 b

Values are means ± standard deviation, and letters following values indicate only the level of Duncan’s analysis for values in the same column (*p* < 0.05).

## 4 Discussion

Many studies have reported that microorganisms isolated from soil environments with the same or similar environments could control related plant disease effectively (Gill and Hyman, 2010; Abdelsattar et al., 2022; Ali et al., 2023). Because the soil in tobacco fields contains bacteria and phage, the BW pathogen and its specific lytic phage were isolated from the rhizosphere soil of tobacco plants in Ningxiang, Hunan Province, and then applied to this area. A good effect in controlling tobacco BW disease was observed. It is important to control outbreaks of tobacco BW to control the spread and migration of the BW pathogen to other crops in this area. It is also significant to provide guidance for scientific isolation, screening, and phage use to control crop bacterial diseases (Holtappels et al., 2021).

The possible adverse environment factors of phage application as a biological agent for plant disease control are mainly temperature, pH, and ultraviolet light. Many studies have confirmed that these factors have a significant effect on the activity of phages (Gill and Hyman, 2010; Abdelsattar et al., 2022). PQ43W showed a strong thermal and pH stability at temperatures no more than 50°C (Figure 2A), with a pH range of 5 to 8 (Figure 2B). However, the UV stability was poor (Figure 2C), which means the environment of phage agents on application or storage should avoid ultraviolet irradiation. The lysis period of phage PQ43W was about 150 min and the burst size was about 74 PFu/cell, which is conducive to the rapid killing of the target bacterial (Figure 2D). Although the biological characteristics of PQ43W are not outstanding, and they are similar to *Ralstonia* phage vB_RsoP_BMB50 (Wang et al., 2022) and *Ralstonia* phage RsPod1EGY (Elhalag et al., 2018), its characteristic of temperatures no more than 50°C, wide pH range stability, and fast lysis capacity make it suitable for most plant-growing environments. This experiment has also enriched phage resource research in controlling crop BW disease.

Because of the phage advantages of specificity, high efficiency, and specificity, it can efficiently eliminate a certain type of bacteria in the environment without affecting other microorganisms (Gill and Hyman, 2010; Hatfull et al., 2022). However, several environmental safety risks should be considered in phage application, especially the transfer or drift of antibiotic resistance genes, virulence genes, and other genetic factors (Liu et al., 2022; Zalewska-Piątek, 2023). The genomic analysis results showed that there was no tRNA, antibiotic resistance, virulence, or solubility genes contained in the PQ43W genome (Table 2). So, PQ43W is a good phage resource in terms of environmental safety.

Some studies reported that the dosage and timings of phage agents could affect the effectiveness of phage therapy (Balogh et al., 2010; Ye et al., 2019). Similarly, the results of the pot experiment showed that phage PQ43W had a higher suppression effect on tobacco BW in higher titer application groups, especially the T1 group (10^8^ PFU/ml), which had a 97.5% control effect on tobacco BW suppression within 30 days, and which was significantly higher than the 200-fold diluted group T200 (26.74%) (*p* < 0.05) (Supplementary Figure S5; Table 3). The field experiment in this study showed consistently that PQ43W had a significant suppression effect on tobacco BW disease compared with the conventional bactericide Zhongshengmycin (Table 5). In addition, Álvarez et al. (2019) demonstrated that phages used through irrigated water to control tomato BW could significantly reduce the number of pathogens within hours. This is because the presence of water is conducive to the spread and migration of phage. Therefore, diluting or adding water for irrigation to provide a water-rich environment may be more effective in controlling plant bacterial diseases.

In this study, PQ43W showed a good effect in controlling tobacco BW disease in pot and field experiments. Considering the diversity of pathogens at species taxonomy classification under natural conditions, the complexity of pathogenic factors, and the diversity of climatic and environmental factors, in order to enhance the effect of phage in controlling crop bacterial disease, “phage cocktail therapy” should be employed, which is the combined use of phages with beneficial microorganisms and / or other measures, such as cultivars, agronomic practice, and soil disinfection. This method is considered to be more effective in scientific control of crop bacterial disease outbreaks (Kumar et al., 2018; Wang X. et al., 2019; Yang et al., 2023).

## 5 Conclusion

In this study, a bacteriophage, PQ43W, was isolated from the rhizosphere soil of tobacco bacterial wilt (BW) disease plants. It was shown to specifically lyse the tobacco BW pathogen *R. ps.* NdE, a member of Rssc. PQ43W had good thermal and pH stability between 30°C and 50°C and pH range 4 to 8 but a poor UV radiation stability. One-step growth curve revealed PQ43W had a short latent period of 15 min and 74 PFU/cell of brust sizes. Sequencing revealed phage PQ43W contained a circular double-stranded DNA genome of 47,156 bp with 65 predicted open reading frames (ORFs), with no tRNA, antibiotic resistance, or virulence genes annotated. Taxonomic classification revealed it belonged to a novel genus of subfamily *Kantovirinae* under *Caudoviricetes*. The phage therapy of PQ43W in pot and field experiments proved that PQ43W is an effective phage when used via non-invasive root irrigation for controlling BW diseases, especially for tobacco cultivation.

## Data availability statement

The original contributions presented in the study are included in the article/Supplementary material, further inquiries can be directed to the corresponding authors.

## Author contributions

BH: Methodology, Writing – original draft, Writing – review & editing. LG: Methodology, Validation, Writing – original draft. DX: Investigation, Methodology, Resources, Writing – original draft. GT: Investigation, Methodology, Writing – original draft. LL: Investigation, Methodology, Writing – original draft. LY: Investigation, Methodology, Writing – original draft. YJ: Investigation, Methodology, Writing – original draft. QL: Conceptualization, Methodology, Supervision, Writing – original draft, Writing – review & editing. WC: Methodology, Writing – original draft, Writing – review & editing. YL: Data curation, Visualization, Writing – original draft. HH: Methodology, Writing – original draft. HS: Data curation, Writing – review & editing. QP: Methodology, Resources, Supervision, Writing – original draft, Writing – review & editing. KY: Funding acquisition, Supervision, Writing – original draft, Writing – review & editing.
