# Supplementary material for: Isolation, characterization, and genomic analysis of a lytic bacteriophage, PQ43W, with the potential of controlling bacterial wilt
Source: Front Microbiol. 2024 Aug 1;15:1396213. doi: 10.3389/fmicb.2024.1396213 (PMC11324598; doi:10.3389/fmicb.2024.1396213)
Supplement: Supplementary file 1 [file Data_Sheet_1.docx]

**Isolation, characterization, and genomic analysis of a lytic bacteriophage, PQ43W, with the potential of controlling bacterial wilt**

Bin. *et al*.

Supplementary Figures S1-S5.


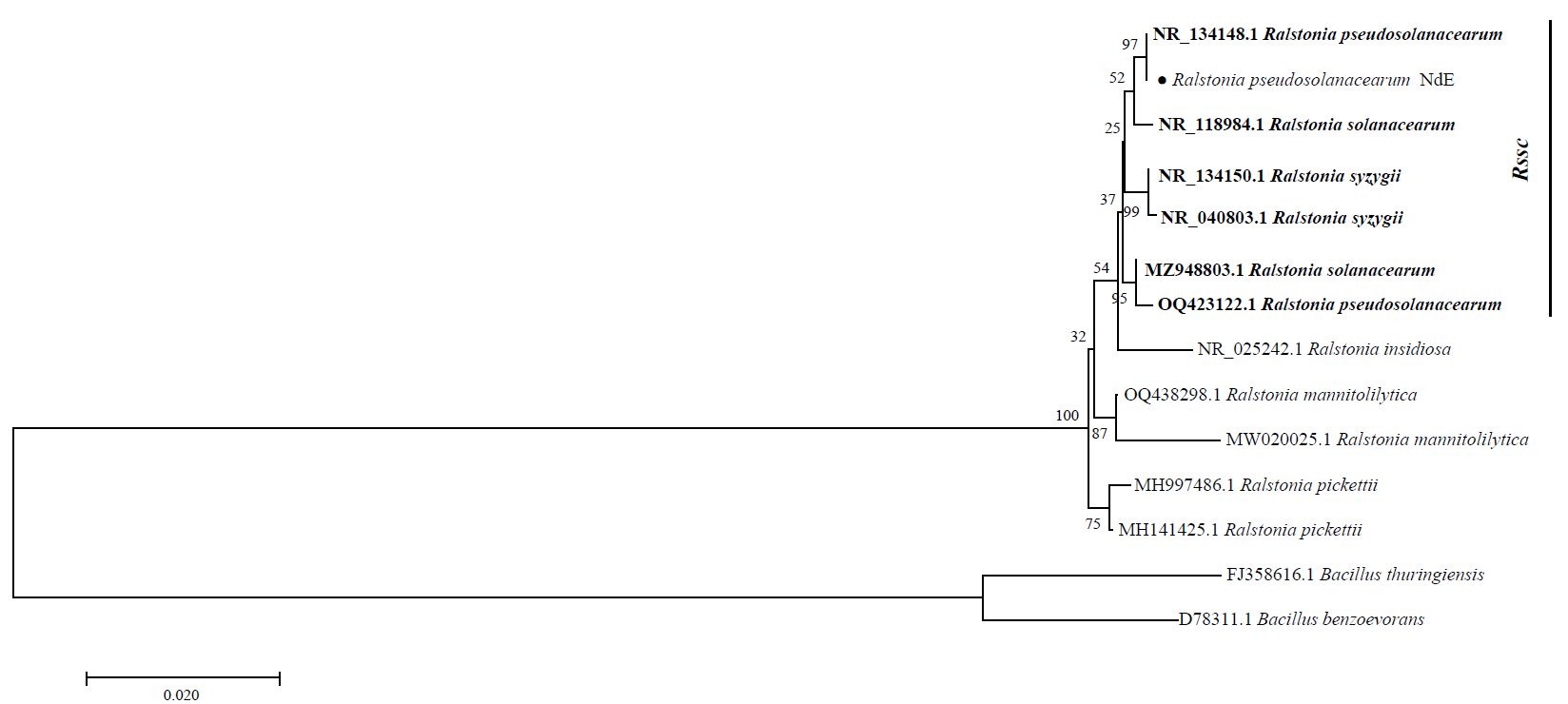


**Supplementary** **Figure S1.** The phylogenetic trees were constructed using neighbor-joining method (NJ) method by MEGA with 16S ribosomal RNA sequence. The horizontal branches are proportional to genetic distance, The Rssc were showed in bold and the strain NdE was marked with black dot.


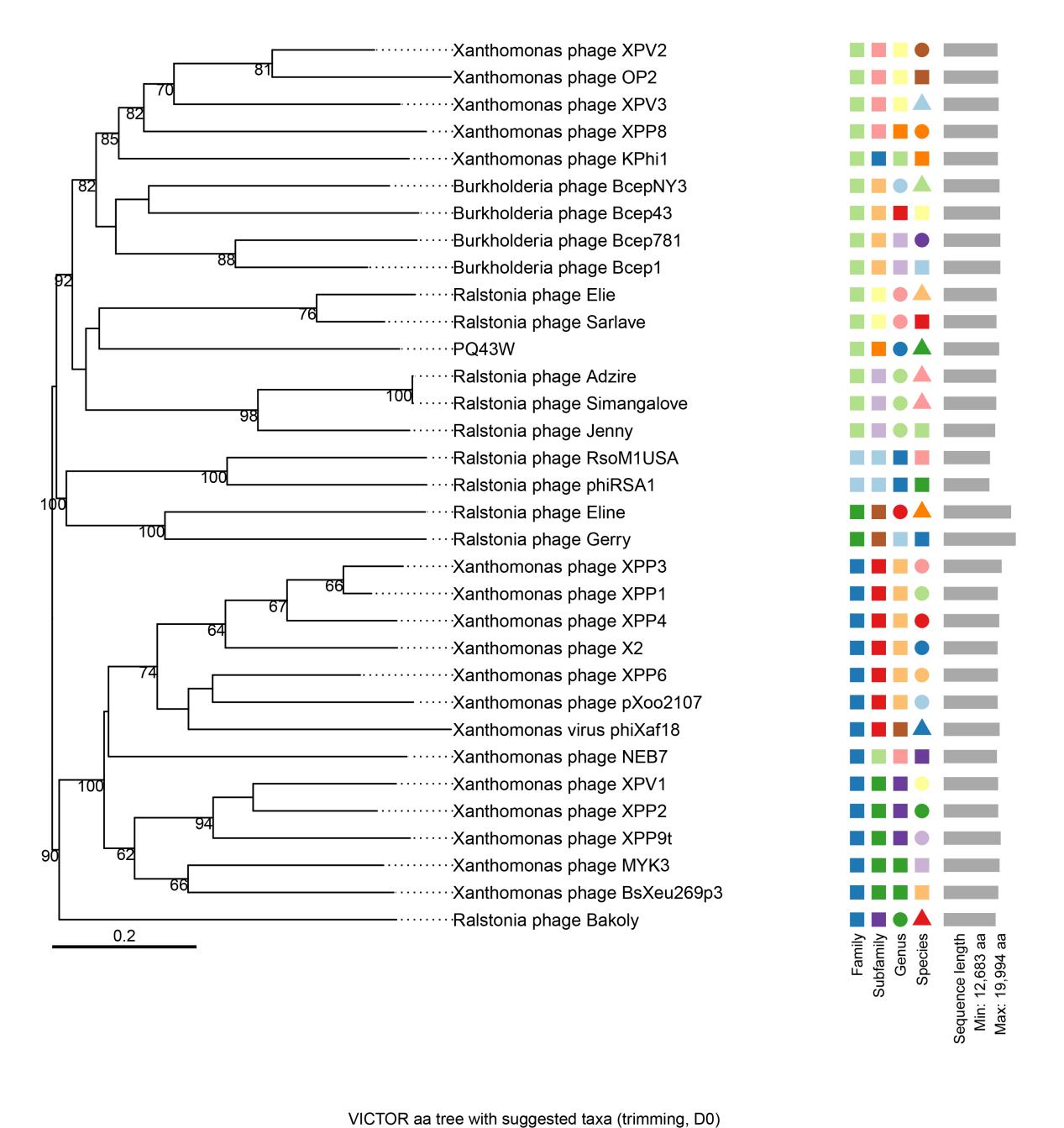


**Supplementary Figure S2.** Phylogenomic tree of PQ43W and its related 32 phages analysis by VICTOR with formula D0. This tree was generated using the Genome-BLAST distance phylogeny (GBDP) method, and the number near each node is the GBDP pseudo-bootstrap support value from 100 replications (only values > 50% are shown). Bacteriophage genus assignments according to the official ICTV classification (March 2023) are provided with different color frames. The GC content and sequence length of each phage genome is indicated on the right.


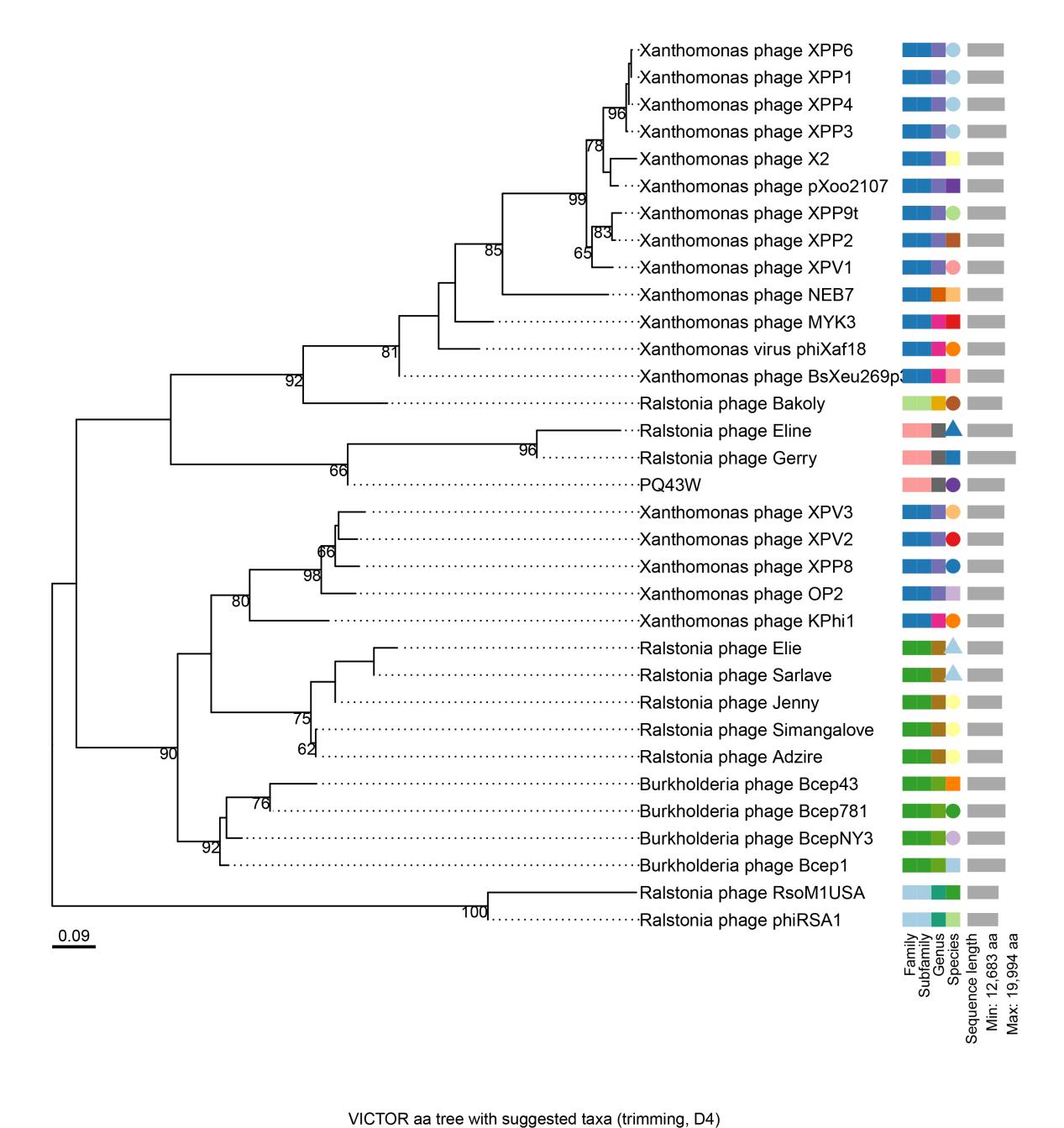


**Supplementary Figure S3.** Phylogenomic tree of PQ43W and its related 32 phages analysis by VICTOR with formula D4. This tree was generated using the Genome-BLAST distance phylogeny (GBDP) method, and the number near each node is the GBDP pseudo-bootstrap support value from 100 replications (only values > 50% are shown). Bacteriophage genus assignments according to the official ICTV classification (March 2023) are provided with different color frames. The GC content and sequence length of each phage genome is indicated on the right.


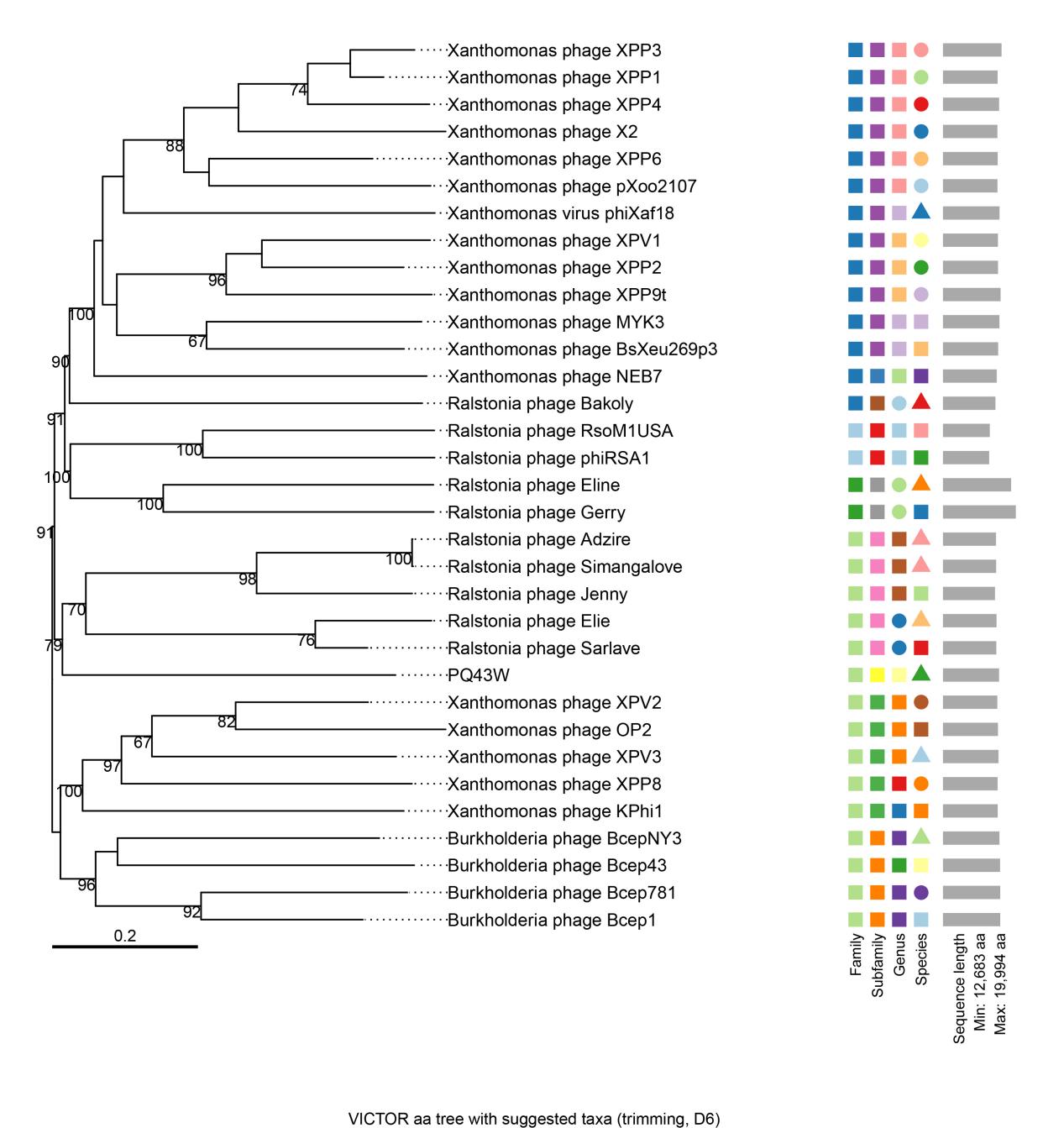


**Supplementary Figure S4.** Phylogenomic tree of PQ43W and its related 32 phages analysis by VICTOR with formula D6. This tree was generated using the Genome-BLAST distance phylogeny (GBDP) method, and the number near each node is the GBDP pseudo-bootstrap support value from 100 replications (only values > 50% are shown). Bacteriophage genus assignments according to the official ICTV classification (March 2023) are provided with different color frames. The GC content and sequence length of each phage genome is indicated on the right.


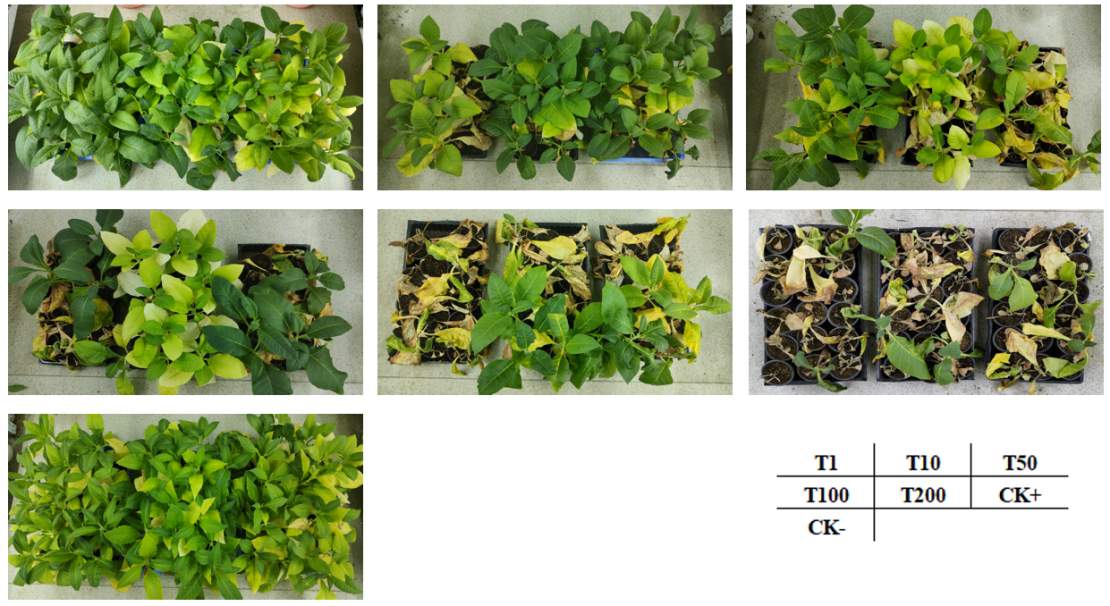


**Supplementary Figure S5.** Effect of PQ43W on control the tobacco bacterial wilt caused by *R. ps.* NdE in pot experiment.
